# MicroRNA-155 expression is independently predictive of outcome in chordoma

**DOI:** 10.18632/oncotarget.3273

**Published:** 2015-03-16

**Authors:** Eiji Osaka, Andrew D. Kelly, Dimitrios Spentzos, Edwin Choy, Xiaoqian Yang, Jacson K. Shen, Pei Yang, Henry J. Mankin, Francis J. Hornicek, Zhenfeng Duan

**Affiliations:** ^1^ Sarcoma Biology Laboratory, Department of Orthopaedic Surgery, Massachusetts General Hospital, Boston, MA 02114, USA; ^2^ Department of Orthopaedic Surgery, Nihon University School of Medicine, Tokyo 173–8610, Japan; ^3^ Fels Institute for Cancer Research & Molecular Biology, Temple University School of Medicine, Philadelphia, PA 19140, USA; ^4^ Division of Hematology/Oncology, Sarcoma Program, Department of Medicine, Beth Israel Deaconess Medical Center, Boston, MA 02215, USA

**Keywords:** miRNA-155 (miR-155), chordoma, prognosis, miRNA microarray assay, RT-PCR

## Abstract

**Background:**

Chordoma pathogenesis remains poorly understood. In this study, we aimed to evaluate the relationships between microRNA-155 (miR-155) expression and the clinicopathological features of chordoma patients, and to evaluate the functional role of miR-155 in chordoma.

**Methods:**

The miRNA expression profiles were analyzed using miRNA microarray assays. Regulatory activity of miR-155 was assessed using bioinformatic tools. miR-155 expression levels were validated by reverse transcription-polymerase chain reaction. The relationships between miR-155 expression and the clinicopathological features of chordoma patients were analyzed. Proliferative, migratory and invasive activities were assessed by MTT, wound healing, and Matrigel invasion assays, respectively.

**Results:**

The miRNA microarray assay revealed miR-155 to be highly expressed and biologically active in chordoma. miR-155 expression in chordoma tissues was significantly elevated, and this expression correlated significantly with disease stage (*p* = 0.036) and the presence of metastasis (*p* = 0.035). miR-155 expression also correlated significantly with poor outcomes for chordoma patients (hazard ratio, 5.32; *p* = 0.045). Inhibition of miR-155 expression suppressed proliferation, and the migratory and invasive activities of chordoma cells.

**Conclusions:**

We have shown miR-155 expression to independently affect prognosis in chordoma. These results collectively indicate that miR-155 expression may serve not only as a prognostic marker, but also as a potential therapeutic target in chordoma.

## INTRODUCTION

Chordoma is a rare, low-grade bone cancer arising from benign notochordal rests [1]. The majority of chordomas arise in the sacrococcygeal region, with the peak incidence occurring between the fifth and sixth decades of life. The main symptom is variable pain, which depends largely on tumor location. Chordoma develops asymptomatically with local invasion and slow growth. Therefore, chordomas are often diagnosed at advanced stages of disease [2]. Chordomas are chemo-resistant and relatively radio-resistant. Thus, the optimal treatment for chordoma is wide surgical resection, which has been shown to decrease local recurrence and metastasis [3–5]. However, it is frequently difficult to adequately achieve negative surgical margins because of tumor location and adjacent critical vital structures. As a result, more than 40% of patients with chordoma experience local recurrence. Moreover, chordomas can metastasize several years after the initial diagnosis, with 10–50% of patients developing metastases during follow-up. Five- and ten-year overall survival rates were 45–77% and 28–50%, respectively [5]. Hence, there is an unmet need to explore new prognostic markers and therapeutic approaches for chordoma patients.

microRNAs (miRs) are small, non-coding RNAs which, via translation inhibition or transcript degradation, regulate many essential biological functions, including development, cell growth, cell proliferation, cell cycle distribution, differentiation, apoptosis, and metabolism [6–9]. It is noteworthy that miRs are thought to be involved in the regulation of 30% of the human genome [8]. Not surprisingly, miR dysregulation correlates with tumorigenesis processes in many cancers including chordoma [10–13], and can serve as either oncogenes or tumor suppressors [7].

microRNA-155 (miR-155) can act as a multifunctional miR, with roles in hematopoiesis, inflammation, immunity, viral infection, cardiovascular disease, and neoplastic diseases [9, 14]. miR-155 is reportedly involved in the tumorigenesis processes of various cancers, and its expression level correlates with poor outcome. However, there have been no reports to date implicating miR-155 in chordoma development.

The aims of this study were to evaluate the relationships between the miR-155 expression level and the clinicopathological features and outcomes of chordoma patients, and to evaluate the functional role of miR-155 expression in a chordoma cell line. We found that miR-155 expression independently affects prognosis in chordoma. Inhibition of miR-155 suppressed not only cell proliferation, but also migratory and invasive activities in chordoma.

## RESULTS

### Global miR expression and activity analysis suggest miR-155 is biologically active in chordoma

The impetus to study the role of miR-155 in chordoma derives from miR microarray-based examination of two frozen clinical chordoma specimens, two chordoma cell lines (U-CH1 and CH8), and two normal human skeletal muscle samples as comparators. The use of skeletal muscle as a control is justified by prior tumor profiling studies in chordoma, and the unresolved question of the true chordoma cell of origin [10, 15–18]. In this preliminary analysis, 67 miRs were found to be significantly differentially expressed between chordoma samples (cell lines and clinical specimens) and normal skeletal muscle (*p* < 0.05; Supplementary Table S1). To identify those miRs most likely to be biologically active in chordoma, we then performed an independent analysis of miR regulatory activity which utilizes mRNA expression data in conjunction with miR target transcript prediction algorithms to infer the relative biological action of specific miRs. Using multiple miR target prediction algorithms (Pictar, Pita, and miRanda), miR-155 was found to have increased regulatory activity in chordoma samples relative to normal tissue (*p* < 0.01, false discovery rate (FDR) < 0.05; Supplementary Table S2). Because miR-155 was specifically found to have higher expression in chordoma tissue relative to normal (*p* = 0.018; Supplementary Table S1, S2), and its role has been characterized in other malignancies, we chose to further investigate its biological relevance in chordoma.

### Overexpression of miR-155 in chordoma

**Figure 1:**
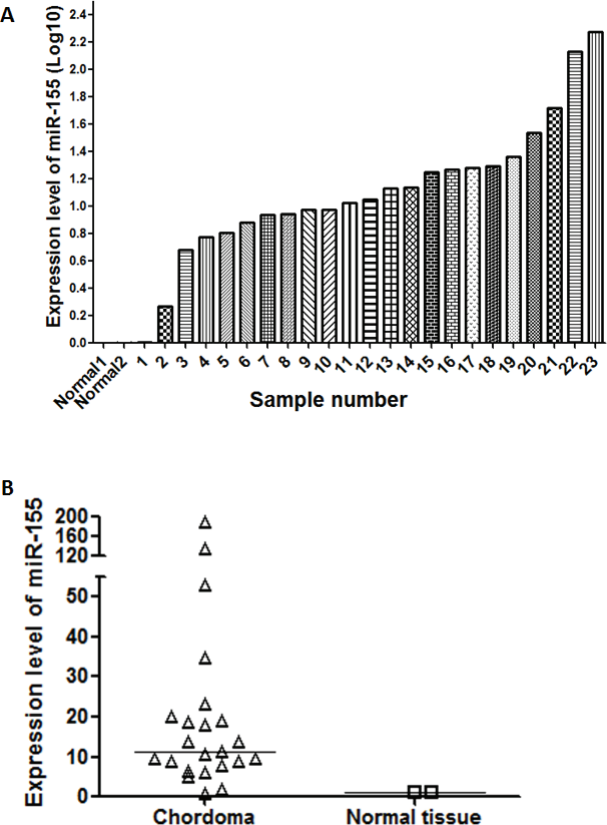
miR-155 is overexpressed in chordoma **(A)** Bar chart showing the distribution of miR-155 expression across 23 clinical chordoma specimens and two normal tissue controls. **(B)** Aggregated expression levels of miR-155 in chordoma samples compared to normal controls.

miR-155 expression was measured in 23 chordoma tissue samples and 2 normal skeletal muscle samples using quantitative reverse transcription-polymerase chain reaction (RT-PCR). All samples expressed miR-155, with expression being substantially higher in chordoma tissues compared to the normal controls (Fig. 1A). The median value of miR-155 expression in chordoma tissues was 11.3-fold that of normal tissues (Fig. 1B).

### Relationships of miR-155 expression level with clinicopathological features of chordoma patients

**Figure 2:**
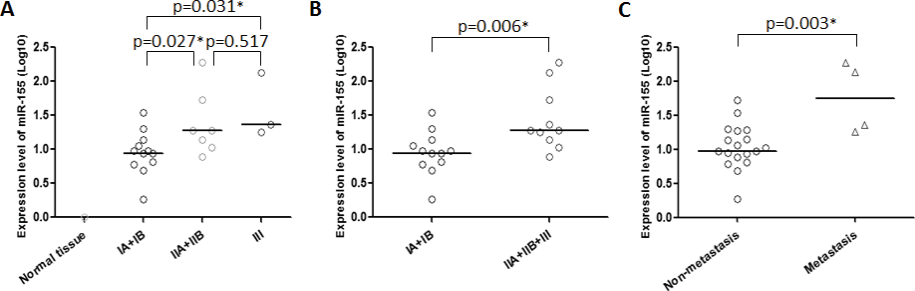
miR-155 expression is correlated with stage and metastatic potential in chordoma Dot-plots showing the relationships between miR-155 expression and **(A, B)** Enneking stage, and **(C)** the presence of metastasis. *p < 0.05.

We evaluated the relationships between miR-155 expression levels and clinicopathological features of chordoma patients including gender, age, location, Enneking stage, origin, the presence of local recurrence, and metastasis. In this analysis “primary origin” was defined as those patients who had never received prior treatment for malignant chordoma, and “recurrent origin” was defined as the subset of patients who presented with recurrent disease after surgery at an outside institution. Among the clinical characteristics evaluated, miR-155 expression was significantly correlated with Enneking stage (*p* = 0.036) and the presence of metastasis (*p* = 0.035) (Table 1). Interestingly, miR-155 expression levels were positively correlated with disease stage in a step-wise manner, and there were significant differences between Stage 1A + 1B and both Stage 2A + 2B and Stage 3 (*p* = 0.027, *p* = 0.031, respectively, Fig. 2A), suggesting that both extent of malignant invasion, and degree of pathologic cellular atypia may be associated with aberrant miR-155 expression. Although there were no statistically significant differences in other clinicopathological features, there was a notable trend for higher miR-155 expression among patients with recurrent disease (Table 1). We also compared quantitative miR-155 expression levels across clinical characteristics and, as expected, we found significant differences associated with Enneking stage (*p* = 0.006) and the presence of metastasis (*p* = 0.003) (Fig. 2B, 2C, Supplementary Table S3).

**Table 1:** Correlation between the expression level of miR-155 with clinicopathological features

Clinicopathological features	Low miR-155	High miR-155	Total	*P*-value
**Gender**				
Male	9	9	18	0.692
Female	2	3	5	
**Age**				
≥ 63	6	6	12	0.827
< 62	5	6	11	
**Location**				
Sacrum	10	10	20	0.59
Other	1	2	3	
**Stage**				
1A + 1B	9	4	13	0.036^a^
2A + 2B + 3	2	8	10	
**Origin**				
Primary origin	8	4	12	0.059
Recurrent origin	3	8	11	
**Local recurrence**				
Overall				
Yes	5	6	11	0.827
No	6	6	12	
Primary origin				
Yes	3	0	3	0.157
No	5	4	9	
Recurrent origin				
Yes	2	6	8	0.782
No	1	2	3	
**Metastasis**				
Yes	0	4	4	0.035^a^
No	11	8	19	

The relationships of the high and low expression group of miR-155 with clinicopathological features of chordoma patients were analyzed by chi-squared test.

a Indicates statistical significance. Statistical significance was defined as a *P*-value of < 0.05.

### Relationship between miR-155 expression and outcomes of chordoma patients

**Figure 3:**
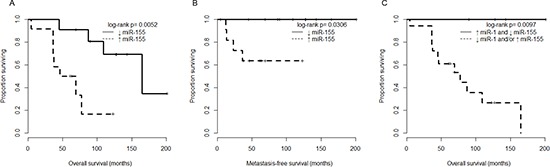
miR-155 expression is associated with overall survival in chordoma patients **(A)** Kaplan-Meier analysis comparing overall survival between chordoma patients with miR-155 expression below the median value (↓ miR-155 group), and chordoma patients with at least median miR-155 expression (↑ miR-155 group). **(B)** Kaplan-Meier analysis comparing metastasis-free survival of chordoma patients stratified as in (A). **(C)** Kaplan-Meier analysis comparing overall survival of chordoma patients stratified by both miR-1 and miR-155 expression (using median expression level cutoffs for each miRNA separately).

We next examined whether clinicopathological features including high and low miR-155 expression levels were associated with poor outcomes in chordoma patients. Based on Kaplan-Meier analysis there were significant differences between the high and low expression groups in overall survival (*p* = 0.0052, Fig. 3A), and metastasis-free survival (*p* = 0.0306, Fig. 3B). Because we had previously observed a positive correlation between miR-1 expression and outcome in chordoma [13], we attempted to combine it with miR-155 expression to improve risk stratification. Consistent with our hypothesis, patients with low miR-155 expression and high miR-1 expression had uniformly favorable overall survival (*p* = 0.0097, Fig. 3C) and the survival discrimination using the combined miR-155/miR-1 analysis appears stronger than that obtained by using any of the two markers individually. We also examined other clinical characteristics in our cohort, and found that, consistent with previous studies, the presence of metastasis significantly affected overall survival (*p* = 0.0015, Fig. S1A) [3–5]. In addition, although it did not reach statistical significance due to small sample size, patients with local recurrence group tended to have inferior outcomes compared to patients with non local recurrence (*p* = 0.0646, Fig. S1B). In a univariate Cox regression analysis, high miR-155 expression and recurrent origin were significantly associated with shorter overall survival of chordoma patents (hazard ratio, 6.35; *p* = 0.011, hazard ratio, 9.77; *p* = 0.004, respectively), whereas other clinicopathological features showed no prognostic correlations (Table 2A). Importantly, multivariate Cox regression revealed that miR-155 expression is significantly associated with shorter overall survival independent of recurrent origin (hazard ratio, 5.32; *p* = 0.045, Table 2B). These results demonstrated the miR-155 expression level to independently predict chordoma outcomes.

**Table 2:** Prognostic factors for overall survival in chordoma

A) Univariate anaysis for prognostic factors of chordoma			
	HR	95% CI	*P*-value
miR-155 expression (High or Low)^a^	6.35	1.54–26.29	0.011^b^
Gender	0.63	0.17–2.40	0.503
Age	1.05	1.00–1.11	0.064
Location	0.61	0.13–2.83	0.524
Stage	1.63	0.71–3.73	0.250
Margin	1.41	0.92–2.15	0.116
Radiation	1.50	0.40–5.61	0.543
Origin	9.77	2.10–45.41	0.004^b^

B) Multivariate anaysis for prognostic factors of chordoma			
miR-155 expression (High or Low)^a^	5.32	1.04–27.36	0.045^b^
Origin	8.28	1.69–40.65	0.009^b^

a The cut-off level was set at the median value of the miR-155 expression levels in 23 patients with chordoma.

b Indicates statistical significance. Statistical significance was defined as a *P*-value of < 0.05.

### Modulating expression levels of miR-155 in a chordoma cell line

**Figure 4:**
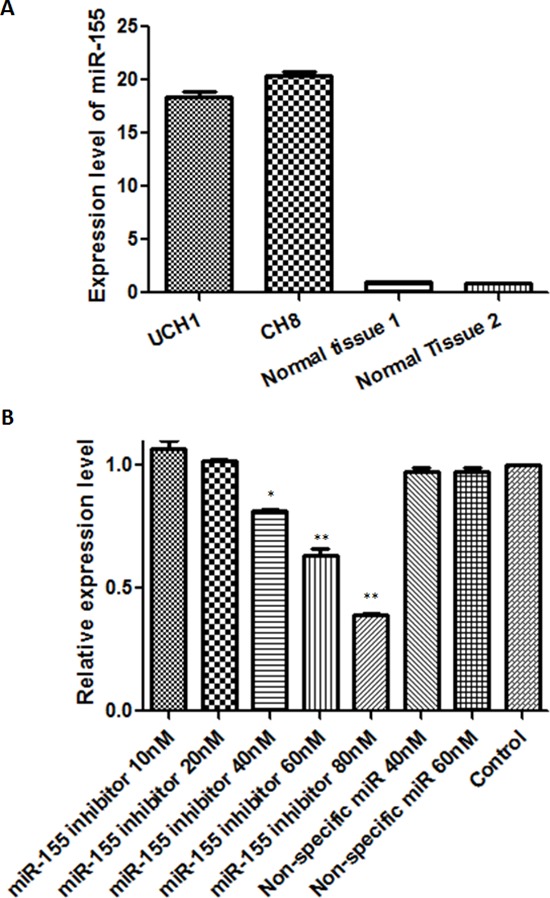
*In vitro* antagonism of miR-155 is feasible in chordoma cell lines **(A)** miR-155 expression levels in U-CH1 and CH8 chordoma cell lines, and normal control tissues. **(B)** miR-155 expression levels after transfection with miR-155 inhibitor at 10–80 nM or non-specific miR inhibitor at 40 nM and 60 nM. *p < 0.05, **p < 0.01 (Comparison of miR-155 inhibitor transfected cells with non-specific miR inhibitor transfected cells or control cells using Student's *t*-test).

Because of the striking association between miR-155 and outcome in clinical specimens, we next sought to examine the effects of modulating miR-155 expression *in vitro*. We first performed experiments to prove the feasibility of miR-155 inhibition using a transfected antagonist. Using quantitative RT-PCR we found miR-155 expression levels in the U-CH1 and CH8 cell lines to be 18.8 and 20.4-fold that of normal tissues, respectively (Fig. 4A). The cells were then transfected with a specific miR-155 inhibitor at increasing concentrations (10 nM, 20 nM, 40 nM, 60 nM, and 80 nM). The miR-155 inhibitor significantly and dose-dependently inhibited miR-155 expression in transfected cells, whereas the non-specific miR inhibitor transfected cells showed no change in miR-155 expression (Fig. 4B). Thus, we had established that modulation of miR-155 expression *in vitro* is feasible for downstream interrogation of its functional consequences.

### Inhibition of miR-155 expression affects the proliferation of chordoma cell lines

**Figure 5:**
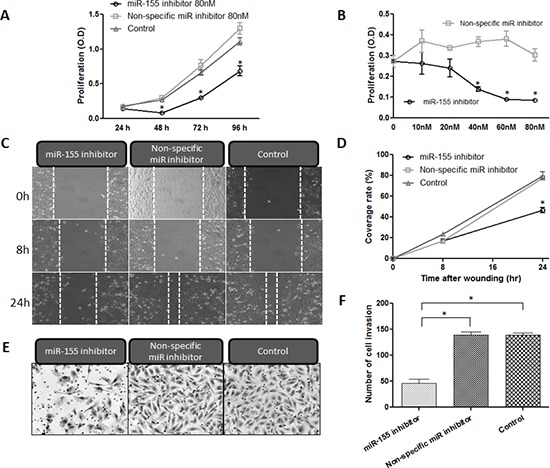
Inhibition of miR-155 decreases proliferation, migration, and invasion of chordoma cells **(A, B)** Cell proliferation assay results for U-CH1 cells transfected with miR-155 inhibitor at concentrations between 0 nM and 80 nM, and incubated for 24–96 h. **(C, D)** miR-155 antagonism resulted in the inhibition of the migratory activity of U-CH1 chordoma cells after transfection with either miR-155 or non-specific miR inhibitor at 40 nM. (C) micrographs of chordoma U-CH1 cells at 0 h, 8 h and 24 h after wounding. (D) The coverage rate of U-CH1 cells for each time point and condition in the wound healing assay. **(E, F)** Invasive activity assay results for U-CH1 cells transfected with miR-155 inhibitor. (E) Micrographs of chordoma U-CH1 cells transfected with 40 nM miR-155 inhibitor. (F) Average numbers of invasive chordoma cells among those transfected with 10–80 nM miR-155. *p < 0.01.

To examine whether down-regulation of miR-155 inhibits the growth of chordoma cells, we conducted Methyl thiazolyl tetrazorium (MTT) assays to evaluate altered *in vitro* proliferation. Chordoma cells were transfected with the miR-155 inhibitor at various concentrations (10 nM, 20 nM, 40 nM, 60 nM, and 80 nM) for 24–96 h. The MTT assays revealed that miR-155 inhibitor transfected chordoma cells showed time-dependent inhibition of cellular growth at the concentrations tested, whereas non-specific miR inhibitor transfected cells and control cells continued to grow for the duration of the observation period (Fig. 5A). After transfection with miR-155 inhibitor or non-specific miR inhibitor at a different concentration and incubation for 72 h, the miR-155 inhibitor transfected cells showed significant and dose-dependent inhibition of cellular growth (Fig. 5B). These results demonstrated that down-regulation of miR-155 inhibits cell proliferation both dose- and time-dependently in chordoma cell lines *in vitro*.

### Aberrant miR-155 expression affects the migratory activities of chordoma cell lines

To assess whether miR-155 inhibitor transfected chordoma cells show altered motility, we conducted wound healing assays to assess migratory activity. We observed the wound area at 0, 8 and 24 h after scratch injury (Fig. 5C). After 24 h incubation, the miR-155 inhibitor transfected cells covered only 46.4% of the scratch defect, whereas the non-specific miR inhibitor transfected cells covered 77.5%. The cell migratory activities were therefore markedly decreased in miR-155 transfected cells as compared with non-specific miR inhibitor transfected cells and control cells (*p* < 0.01, Fig. 5D).

### Inhibition of miR-155 suppressed the invasive activities of chordoma cells

We next assessed whether miR-155 inhibitor transfected chordoma cells showed altered invasive activity using a matrigel invasion assay. After transfection, the matrigel invasion assay showed that transfection with the miR-155 inhibitor resulted in marked inhibition of the invasive activities of chordoma cells (Fig. 5E). The number of invasive cells among the transfected chordoma cells was decreased to less than 32.9% of that in the control chordoma cells (*p* < 0.01, Fig. 5F).

## DISCUSSION

miRNAs are small RNA molecules which regulate the expression of many genes primarily at the transcript level, and are expressed in a highly tissue-specific manner. Aberrant miR expression has now been shown to affect a number of pathways important for cancer initiation and maintenance in many malignancies including chordoma. The dysregulated expression of several miRs including miR-1, miR-206, miR-31, miR-222, miR-140–3p, and miR-148a has been associated with chordoma in recent literature, however there is still a dearth of molecular biomarkers suitable for clinical application in this disease [10, 11, 13]. In this study, our effort to address this unmet need revealed for the first time using miR and mRNA microarrays and miR regulatory activity analysis that miR-155 expression and biological activity is significantly elevated in chordoma relative to normal tissues. These observations are consistent with recent data in other tumor settings showing that miR-155 can functionally act as an oncogene, and that its targets are involved in pathways regulating proliferation, apoptosis, differentiation, angiogenesis, and epithelial-to-mesenchymal transition [7, 9, 14, 19]. In addition, observed miR-155 upregulation in several cancers has been associated with poor outcomes [20, 21]. Our study presents the first data demonstrating that miR-155 is upregulated in chordoma, and that miR-155 expression is correlated with both advanced disease stage and the presence of metastasis. Our data further show that high miR-155 expression is associated with shorter overall survival independent of other prognostic factors. Based on our prior observations relating miR-1 expression to outcome in chordoma, we also tested whether a combined model could improve risk stratification, and found that patients with both high miR-1 and low miR-155 had strikingly favorable outcomes, suggesting that a dual miRNA panel may provide even greater clinical utility.

Tumor growth, migratory, and invasive abilities in cancers including chordoma are required for initiation of the metastatic process. Recent studies in other malignancies have shown that miR-155 correlates with diverse biological functions, including proliferation, migration and invasion, apoptosis, and cell cycle progression [7, 9, 14, 19]. In the present study, we demonstrated that controlled down-regulation of miR-155 expression inhibits multiple *in vitro* correlates of tumor aggression including cell proliferation, and both migratory and invasive abilities in chordoma. These data suggest that modulating miR-155 expression could plausibly control tumor progression, and inhibit local recurrence and metastasis in chordoma. Given the degree to which chordomas are typically refractory to standard therapies, miR-155 presents an exciting and novel avenue to potentially improve clinical outcomes.

At present, the reasons underlying miR-155-mediated aggression in chordoma remain unclear, although recent data provide some interesting hypotheses. It has been established that each miR can interact with up to 200 potential target genes, making the task of dissecting downstream pathway aberrations challenging [9, 22]. However, various direct targets of miR-155 have been implicated in certain cancer settings. For example, suppressor of cytokine signaling 1 (SOCS1) and tumor protein 53-induced nuclear protein 1 (TP53INP1) – two validated targets of miR-155 –have been shown to regulate tumor growth, invasion, and metastasis [9]. SOCS1 does this via inhibition of signal transducers and activators of transcriptions (STAT3) [23–25]. The plausibility of this mechanism underlying our findings is supported by data implicating STAT3 up-regulation in chordoma [26]. Functional experiments have shown that inhibition of miR-155 induces elevated SOCS1 expression and the subsequent suppression of STAT3 in various cancers [23–25]. In addition to SOCS1, TP53INP1, represents another validated target for miR-155, which is a proapoptotic stress-induced p53 target gene and a known tumor suppressor gene. According to recent literature overexpression of miR-155 can strongly reduce TP53INP1 expression in cancers, and inhibition of miR-155 leads to the suppression of tumor growth *in vitro* and *in vivo* [27–29].

Studies in other cancers have also shown that modulation of miR-155 expression can alter proliferation and invasiveness [21, 25]. Upon transfection with miR-155 inhibitors or mimics cancer cells have shown marked inhibition or promotion of proliferation and invasion, respectively. Table 3 shows predicted direct targets of miR-155 in cancer, many of which are known regulators of pathways important for disease progression, including epithelial-mesenchymal transition (EMT), proliferation, differentiation, apoptosis, and angiogenesis [23, 24, 28–54]. These examples of validated and predicted target genes illustrate how modulation of miR-155 can potentially attenuate tumor progression via multiple mechanisms, and is therefore a promising target for therapeutic intervention in chordoma.

**Table 3:** Predicted direct targets of miR-155 in cancer

Gene name	Official name	Biological functions	Reported in cancer	References
SOCS1	Suppressor of cytokine signaling 1	Cell proliferation, invasion and metastasis	Breast cancer, hepatocellular carcinoma, laryngeal squamous cell carcinoma, B chronic lymphocytic leukemia, colon cancer	[23], [24], [30], [31], [32]
TP53INP1	Tumor protein 53-induced nuclear protein 1	Cell apoptosis, proliferation, invasion, EMT, angiogenesis, metabolism	Brest cancer, pancreatic cancer	[27], [28]
PPP2CA	Protein phosphatase 2, catalytic subunit, alpha isozyme	Cell growth	Colon cancer	[32]
Bcl-2	B-cell lymphoma 2	Cell apoptosis	Leukemic B-cell	[33]
Smad2	Mothers against decapentaplegic homolog 2	Cell migration, invasion, adhesion	Gastric cancer	[34]
Smad5	Mothers against decapentaplegic homolog 5	Cell proliferation, differentiation, apoptosis, metastasis	Lymphoma	[35]
RhoA	Ras homolog gene family, member A	Cell apoptosis, proliferation, invasion, EMT, angiogenesis, metabolism	Breast cancer	[36]
Apaf-1	Apoptotic peptidase activating factor 1	Cell apoptosis	Lung cancer	[37]
SPI1 (PU.1)	Spi-1 proto-oncogene	Transcription	Lymphoma	[38]
C/EBPβ	CCAAT-enhancer-binding protein β	Cell apoptosis, proliferation, invasion, EMT, angiogenesis, metabolism, differentiation	Breast cancer	[39]
VHL	Von Hippel–Lindau	Cell apoptosis, proliferation, invasion, EMT, angiogenesis, metabolism	Breast cancer	[40]
SEL1L	Protein sel-1 homolog 1	Cell proliferation, apoptosis	Pancreatic ductal adenocarcinoma	[41]
JMJD1A	Jumonji domain-containing 1a	A negative prognostic marker	Nasopharyngeal carcinoma	[42]
BACH1	BTB and CNC homology 1	Cell proliferation, invasion, apoptosis	Nasopharyngeal carcinoma, renal cancer	[42], [43]
SHIP1	Src homology 2-containing inositol phosphatase-1	Proliferation, differentiation, apoptosis, metastasis	Chronic lymphocytic leukemia	[44]
HDAC4	Histone deacetylase 4	Cell proliferation, differentiation, apoptosis, metastasis	Lymphoma	[45]
PIK3R1	Phosphoinositide-3-kinase, regulatory subunit 1 (alpha)	Cell proliferation, differentiation, apoptosis, metastasis	Lymphoma	[46]
APC	Adenomatous polyposis coli	Cell growth, proliferation, apoptosis	Papillary thyroid carcinoma, hepatocellular carcinoma	[47], [52]
CLDN1	Claudin 1	Cell growth, proliferation, migration, invasion	Ovarian cancer	[48]
SKI	Ski	Cell growth, proliferation, apoptosis	Melanoma	[49]
CDC73	Cell division cycle 73	Cell proliferation, apoptosis	Oral squamous cell carcinoma	[50]
FOXO3a	Forkhead box O3a	Cell apoptosis, proliferation, invasion, EMT, angiogenesis, metabolism	Breast cancer	[51]
MLH1	MutL homolog 1	Cell proliferation, apoptosis	Pancreatic cancer	[53]
CK1α	Casein kinase-1alpha	Cell proliferation, cell cycle	Liposarcoma	[54]

In summary, our study is the first to show that miR-155 expression is predictive of chordoma outcomes independent of other clinical factors. Additionally, our *in vitro* experiments demonstrated that inhibition of miR-155 suppressed cell proliferation, migratory and invasive activities in a chordoma cell line. Thus, our data suggest that miR-155 expression is not only a potential prognostic marker, but is also a possible therapeutic target in chordoma. Future studies should examine the feasibility of using a miR-155 antagonist with *in vivo* models, and interrogate aberrant downstream signaling pathway activity in chordoma which can be modulated therapeutically with existing small molecules.

## MATERIALS AND METHODS

### Human chordoma tissue samples

Samples from 23 patients who had undergone surgical resection of chordoma were obtained from the Massachusetts General Hospital (MGH) sarcoma tissue bank, and were studied according to the policies of the institutional review board of the hospital. This study was conducted with the approval of the MGH Institutional Review Board (IRB protocol #:2007-P-002464/5). All specimens were assessed by light microscopy and immunohistochemistry. The clinical characteristics of the chordoma patients are presented in Table 4.

**Table 4:** Clinical characteristics of the chordoma patients

Sample number	Age/Gender	Location	Origin^a^	Stage	Margin	Radiation	Local recurrent	Metastasis	Follow up (months)	Status
1	70/M	Sacrum	Recurrent	1B	Intraleasional margin	No	Yes	No	165	DOD
2	71/M	Sacrum	Primary	1B	Wide margin	No	No	No	69	NED
3	77/M	Sacrum	Primary	1B	Wide margin	Yes	No	No	109	DOO
4	46/M	Sacrum	Primary	1B	Wide margin	Yes	Yes	No	127	AWD
5	64/M	Sacrum	Primary	1B	Wide margin	Yes	No	No	90	NED
6	55/M	Sacrum	Primary	2A	Wide margin	Yes	Yes	No	201	AWD
7	62/M	Sacrum	Primary	1B	Wide margin	Yes	No	No	143	NED
8	83/M	Sacrum	Recurrent	1B	Intraleasional margin	Yes	Yes	No	45	DOO
9	71/F	L4	Primary	1B	Attempted wide margin	Yes	No	No	143	NED
10	51/F	Sacrum	Primary	1B	Wide margin	No	Yes	No	87	DOD
11	37/M	Sacrum	Recurrent	2B	Wide margin	No	No	No	128	AWD
12	74/M	Sacrum	Recurrent	1B	Intraleasional margin	Yes	Yes	No	69	DOD
13	58/M	Sacrum	Recurrent	1B	Intraleasional margin	No	Yes	No	37	DOD
14	47/M	Sacrum	Primary	2B	Attempted wide margin	Yes	No	No	123	NED
15	82/M	Sacrum	Recurrent	3	Attempted wide margin	Yes	Yes	Yes	36	DOD
16	63/M	Sacrum	Primary	2A	Wide margin	No	No	No	63	NED
17	63/M	Sacrum	Primary	2A	Wide margin	No	No	No	63	NED
18	60/F	Sacrum	Primary	1A	Wide margin	Yes	No	No	61	NED
19	82/M	Sacrum	Recurrent	3	Attempted wide margin	Yes	Yes	Yes	36	DOD
20	52/F	L3	Recurrent	1B	Wide margin	Yes	No	No	4	DOO
21	52/F	Sacrum	Recurrent	2B	Attempted wide margin	Yes	Yes	No	46	DOD
22	50/M	T12-L1	Recurrent	3	Attempted wide margin	Yes	Yes	Yes	77	DOD
23	74/M	Sacrum	Recurrent	2B	Intraleasional margin	Yes	No	Yes	36	DOD

Abbreviations: L, lumber spine; T, thoracic spine; AWD, alive with disease; NED, no evidence of disease; DOD, dead of disease; DOO, dead of others.

a Origin was defined as patient who was previously treated (recurrent origin) or not (primary origin).

### RNA extraction from cell line and frozen tissue

Total RNA was extracted from the chordoma cell lines and chordoma tissue samples with TRIzol reagent (Invitrogen, CA) by the acid-guanidium-thiocyanate-phenol chloroform method. The total RNA quantity was determined using a SPECTRAmax Microplate Spectrophotometer from Molecular Devices (Sunnyvale, CA) at 490 nm. RNA purity was assessed by the ratio of absorbance at 260/280 nm.

### miR and mRNA expression assays

miR expression was analyzed using miR expression profiling (LC Sciences, TX). 5 μg of total RNA for each sample was fractionated using the Microcon YM-100 (Millipore, MA), and the small RNAs (< 300 nucleotides) were 3′-extended with a poly(A) tail by poly(A) polymerase. miR expression profiling was carried out as previously described [18]. mRNA expression levels for use in miR activity analysis were determined using GeneChip Human Genome U133A 2.0 Array (Affymetrix, CA) performed at the MGH Core Facility.

### miR regulatory activity analysis

miR regulatory activity analysis was performed using an R implementation of the regulatory effects scoring (RE-score) method developed by Cheng et al [55]. In brief, using mRNA expression data, an RE-score was calculated for known miRs using the average difference in expression of miR target transcripts and non-target transcripts. Target transcripts were determined by a user-defined choice of established miR target prediction algorithms including Pita, Pictar, or TargetScan [56–58]. mRNA data were imported into the R environment and miRs were determined to be significantly differentially activated if the RE-score was associated with a Wilcoxon rank sum *p*-value less than 0.05, and an estimated FDR less than 0.05. In this study, a negative RE-score indicates that the associated miR is more active in chordoma specimens than in normal tissue. The script used in this analysis has been uploaded as Supplementary Table S1. An independent gene set analysis (GSA) method was also employed to infer miR activity. This method used miRanda miR target predictions to compare expression levels of sets of miR targets across two phenotypes (chordoma versus normal). GSA for miR target gene sets was implemented on NCI-BRB ArrayTools [59–61].

### TaqMan reverse transcription-PCR for quantification of miR-155

To validate miR expression levels, we performed quantitative RT-PCR for miR-155 (ID 002623, Applied Biosystems, CA) and RNU48 (ID 001006, Applied Biosystems, CA) using the TaqMan^®^ MicroRNA Assay and the TaqMan^®^ Universal PCR Master Mix (Applied Biosystems, CA), followed by evaluation using a StepOnePlus Real time PCR System (Applied Biosystems, CA) according to the manufacturer's instructions. RNU48 miR was used as an endogenous control. The relative gene expression levels were calculated by the 2^ − ΔΔCt method [62], and normalized by RNU48. The relative expression level was log_10_ transformed prior to analysis. All experiments were carried out in triplicate.

### Cell lines and cell cultures

U-CH1, an established human chordoma cell line, was obtained from the University Hospitals of Ulm, Ulm, Germany [63]. Another human chordoma cell line, CH8, was established in our laboratory as previously reported [64]. These cell lines were cultured in RPMI1640 (Invitrogen, CA) supplemented with 10% fetal bovine serum (FBS) and 1% penicillin/streptomycin (Invitrogen, CA) under a humidified incubator of 5% CO_2_-95% air atmosphere at 37°C. The cells were passaged every 3–4 days using trypsin-EDTA until reaching 90% confluence.

### miR-155 inhibitor and transfection

Cells were transiently transfected with Lipofectamine^TM^ RNAiMAX (Invitrogen, CA) according to the manufacturer's instructions. The cells were transfected with various concentrations of miR-155 inhibitor (Ambion^®^Anti-miR^TM^ miRNA Inhibitor, TX) and non-specific miR inhibitor (Ambion^®^ Anti-miR^TM^ miRNA inhibitor Negative Control #1, TX) used as a negative control. The total RNA was harvested 48 h after transfection. The sequence of mature miR for miR-155 is 5′-CUCCUACAUAUUAGCAUUAACA-3′.

### MTT cell proliferation assay

Cell proliferation was evaluated by the MTT assay. Briefly, the cells (2 × 10^3^ cells/well) were seeded into each well of four 96 well plates in antibiotic-free RPMI 1640 supplemented with 10% FBS. The cells transfected with miR-155 inhibitor or non-specific miR inhibitor at various concentrations were incubated for 24–96 h. At a different time point after transfection, 20 μl of MTT (5 mg/ml, Sigma, MO) were added, and the plate was incubated at 37°C for another 4 h. In turn, the MTT formazan was dissolved in acid-isopropanol. Then, the reaction density was read at 490 nm on a SPECTRAmax Microplate Spectrophotometer. The MTT cell proliferation assays were repeated for four days. This experiment was carried out in triplicate.

### Wound healing assay

To evaluate cell migration activity, wound healing assays were performed. Briefly, transfected cells (2 × 10^5^ cells/well) were seeded onto the 12 well plates. After the cells had reached confluence, wounds were made by scraping with a 200 μl tip. After being washed 3 times in serum-free medium, the cells were incubated in regular medium. The wounds at 0, 8 and 24 h were captured by a microscope (Nikon Instruments Inc, NY). The wound areas captured three images per well at different time points (10 × objective), and were calculated employing Image J Software. The coverage rate was represented by subtracting the wound area at each time point from the wound area at the 0 h time point, divided by the wound area at the 0 h time point.

### Matrigel invasion assay

Cell invasion activity was determined by matrigel invasion assay using a BD BioCoat^TM^ Matrigel^TM^ Invasion Chamber (Becton-Dickinson, MA). The matrigel invasion assay was performed according to the manufacturer's instructions. Briefly, transfected cells (5 × 10^4^ cells/well) were seeded onto the upper chamber in the medium without FBS and antibiotics, and medium containing 10% FBS was added to the bottom chambers. Cell invasion was determined after 48 h of incubation. The invading cells were fixed using 100% methanol after removing non-invading cells by scrubbing from the upper membrane with cotton swabs, and in turn, stained with hematoxylin. The invading cell number was counted in three images per membrane under light microscopy (20 × objective).

### Statistical analysis

The relationships between miR-155 expression levels and clinicopathological features of chordoma patients were analyzed by chi-squared test and Student's *t*-test. Using microarray data, significantly differentially expressed miRs were identified by Student's *t*-test. The miR-155 expression levels were divided by the median value. The high expression group was set above the median value, and the low expression group was set below the median value. Overall survival curves were analyzed with the Kaplan–Meier method and compared using the log-rank test. To verify the prognostic factors for overall survival, Cox proportional hazards modeling was performed. To detect independent prognostic factors in multivariate analysis, those factors with significant Cox regression coefficients in univariate analysis were selected. All statistical analyses were carried out using SPSS version 21.0 (SPSS Inc, IL), or the Survival package in R. A statistically significant difference was defined as *p*-value less than 0.05.

## SUPPLEMENTARY TABLES AND FIGURE


