# Supplementary material for: MicroRNA-155 expression is independently predictive of outcome in chordoma
Source: Oncotarget. 2015 Mar 16;6(11):9125–39. doi: 10.18632/oncotarget.3273 (PMC4496207; doi:10.18632/oncotarget.3273)
Supplement: Supplementary file 1 [file oncotarget-06-9125-s001.pdf]

## SUPPLEMENTARY TABLES AND FIGURE

**Supplementary Table S1. miRNA expression profiling in chordoma**

| microRNA | *p*-value | Mean Normal | Mean Chordoma |
|---|---|---|---|
| hsa-miR-1 | 0.001 | 27231.4 | 31.3 |
| hsa-miR-206 | 0.001 | 16049.0 | 29.5 |
| hsa-miR-26a | 0.003 | 35790.6 | 9611.8 |
| hsa-miR-95 | 0.003 | 4243.3 | 18.9 |
| hsa-miR-23a | 0.004 | 21432.1 | 11497.6 |
| hsa-miR-101 | 0.004 | 875.3 | 20.6 |
| hsa-miR-133b | 0.004 | 15708.8 | 68.3 |
| hsa-miR-133a | 0.004 | 16094.8 | 74.7 |
| hsa-miR-1268 | 0.005 | 2042.8 | 134.1 |
| hsa-miR-193a-5p | 0.005 | 283.5 | 2717.0 |
| hsa-miR-27a | 0.006 | 11084.8 | 4992.5 |
| hsa-miR-139-5p | 0.006 | 705.4 | 17.2 |
| hsa-miR-34a | 0.008 | 98.2 | 711.2 |
| hsa-miR-128 | 0.009 | 5141.8 | 616.2 |
| hsa-miR-320c | 0.010 | 787.1 | 8221.0 |
| hsa-miR-320a | 0.011 | 905.5 | 8227.1 |
| hsa-miR-23b | 0.011 | 21411.4 | 10999.0 |
| hsa-miR-221 | 0.012 | 451.9 | 8061.4 |
| hsa-miR-320b | 0.013 | 617.6 | 7706.4 |
| hsa-miR-320d | 0.013 | 475.4 | 6497.5 |
| hsa-miR-199a-5p | 0.015 | 246.1 | 1817.6 |
| hsa-miR-16 | 0.015 | 15115.1 | 5025.6 |
| hsa-miR-214 | 0.016 | 2378.7 | 10784.6 |
| hsa-miR-30b | 0.017 | 8789.7 | 1555.8 |
| hsa-miR-1308 | 0.018 | 327.4 | 6861.4 |
| **hsa-miR-155** | **0.018** | **117.6** | **1359.6** |
| hsa-miR-99b | 0.021 | 445.6 | 2868.4 |
| hsa-let-7a | 0.022 | 34643.8 | 15626.8 |
| hsa-miR-30e | 0.026 | 408.9 | 55.0 |
| hsa-let-7f | 0.028 | 33505.7 | 12725.1 |
| hsa-miR-22 | 0.030 | 863.8 | 135.7 |
| hsa-miR-30c | 0.030 | 7559.2 | 2373.7 |
| hsa-miR-378 | 0.033 | 2735.0 | 159.3 |
| hsa-let-7d | 0.033 | 26905.7 | 13981.6 |
| hsa-miR-130a | 0.033 | 62.0 | 367.5 |
| hsa-miR-140-3p | 0.034 | 281.2 | 17856.1 |

(*Continued*)

| microRNA | *p*-value | Mean Normal | Mean Chordoma |
|---|---|---|---|
| hsa-miR-222 | 0.036 | 557.6 | 6547.4 |
| hsa-miR-126 | 0.039 | 9251.9 | 771.0 |
| hsa-miR-181b | 0.043 | 19.0 | 1143.1 |
| hsa-let-7i | 0.043 | 7727.4 | 19842.1 |
| hsa-miR-572 | 0.002 | 191.4 | 8.4 |
| hsa-miR-933 | 0.003 | 106.7 | 13.3 |
| hsa-miR-940 | 0.003 | 119.0 | 31.8 |
| hsa-miR-1228 | 0.004 | 34.0 | 10.8 |
| hsa-miR-130b | 0.005 | 21.0 | 167.1 |
| hsa-miR-628-3p | 0.009 | 300.9 | 55.2 |
| hsa-miR-30b | 0.011 | 59.8 | 15.4 |
| hsa-let-7f-1 | 0.011 | 66.8 | 17.1 |
| hsa-miR-378 | 0.013 | 191.8 | 13.8 |
| hsa-miR-181d | 0.015 | 6.1 | 103.5 |
| hsa-miR-584 | 0.017 | 28.8 | 84.5 |
| hsa-miR-188-5p | 0.018 | 59.2 | 12.7 |
| hsa-miR-563 | 0.018 | 32.2 | 7.4 |
| hsa-miR-486-3p | 0.019 | 287.4 | 34.2 |
| hsa-miR-542-3p | 0.020 | 55.0 | 8.2 |
| hsa-miR-671-5p | 0.020 | 9.9 | 73.4 |
| hsa-miR-628-5p | 0.023 | 109.4 | 20.0 |
| hsa-miR-148b | 0.023 | 27.7 | 175.7 |
| hsa-miR-484 | 0.025 | 30.8 | 251.7 |
| hsa-miR-125a-3p | 0.025 | 12.6 | 30.2 |
| hsa-miR-126 | 0.028 | 46.0 | 5.6 |
| hsa-miR-489 | 0.029 | 205.4 | 30.9 |
| hsa-miR-1281 | 0.033 | 186.4 | 26.7 |
| hsa-miR-145 | 0.035 | 221.6 | 48.5 |
| hsa-miR-138 | 0.038 | 5.0 | 110.7 |
| hsa-miR-20b | 0.048 | 333.9 | 118.6 |
| hsa-miR-886-5p | 0.049 | 5.3 | 31.9 |

## Supplementary Table S2. Differential expression and regulatory activity of miR-155 in chordoma

| miR-155 expression determined by microarray | | | | |
|---|---|---|---|---|
| Mean Normal Expression | Mean Chordoma Expression | *p*-value | | |
| 117.6 | 1359.6 | 0.018 | | |
| **miR-155 regulatory activity inferred by target mRNA expression** | | | | |
| Target prediction method (RE-score method) | Normalized RE-score | *p*-value | FDR | |
| Pictar | −4.24 | < 0.01 | 0.01 | |
| Pita | −4.09 | < 0.01 | 0.02 | |
| miRanda (GSA method) | LS permutation *p*-value | KS permutation *p*-value | Efron-Tibshirani's GSA test *p*-value | Goeman's global test *p*-value |
| | 0 | 0.04 | 0.2 | 0.01 |

miR-155 expression levels were found to be significantly higher in chordoma relative to normal samples by miRNA microarray. mRNA microarray data were used to infer regulatory activity of miR-155 with two distinct computational techniques which include miRNA target predictions with three distinct algorithms (Pictar, Pita, and miRanda). The RE-score method compares the difference in average expression levels of target genes and non-target genes across two phenotypes. A negative RE-score is indicative of higher regulatory activity in chordoma relative to normal tissue. The GSA method compares expression levels of target gene sets (determined by miRanda) between chordoma and normal tissue with several statistical tests implemented in NCI-BRB Arraytools.

**Supplementary Table S3. Correlation between the expression level of miR-155 in clinicopathological features**

| Clinicopathological features | Total | Average[a] ± SD | *P*-value |
|---|---|---|---|
| **Gender** | | | |
| Male | 18 | 1.30 ± 0.34 | 0.347 |
| Female | 5 | 1.06 ± 0.55 | |
| **Age** | | | |
| ≥ 63 | 12 | 1.01 ± 0.59 | 0.304 |
| < 62 | 11 | 1.23 ± 0.41 | |
| **Location** | | | |
| Sacrum | 20 | 1.05 ± 0.48 | 0.12 |
| Other | 3 | 1.23 ± 0.58 | |
| **Stage** | | | |
| 1A + 1B | 13 | 0.87 ± 0.41 | 0.006[b] |
| 2A + 2B + 3 | 10 | 1.43 ± 0.46 | |
| **Origin** | | | |
| Primary origin | 12 | 0.94 ± 0.30 | 0.088 |
| Recurrent origin | 11 | 1.31 ± 0.64 | |
| **Local recurrence** | | | |
| Overall | | | |
| Yes | 11 | 1.11 ± 0.56 | 0.93 |
| No | 12 | 1.13 ± 0.49 | |
| Primary origin | | | |
| Yes | 3 | 0.88 ± 0.10 | 0.69 |
| No | 9 | 0.96 ± 0.34 | |
| Recurrent origin | | | |
| Yes | 8 | 1.19 ± 0.64 | 0.354 |
| No | 3 | 1.61 ± 0.63 | |
| **Metastasis** | | | |
| Yes | 4 | 1.76 ± 0.52 | 0.003[b] |
| No | 19 | 0.98 ± 0.41 | |

The relationships of quantitative miR-155 expression levels with clinicopathological features of chordoma patients were analyzed by Student's *t*-test.
[a]Log10 transformed
[b]Indicates statistical significance. Statistical significance was defined as a *P*-value of <0.05.

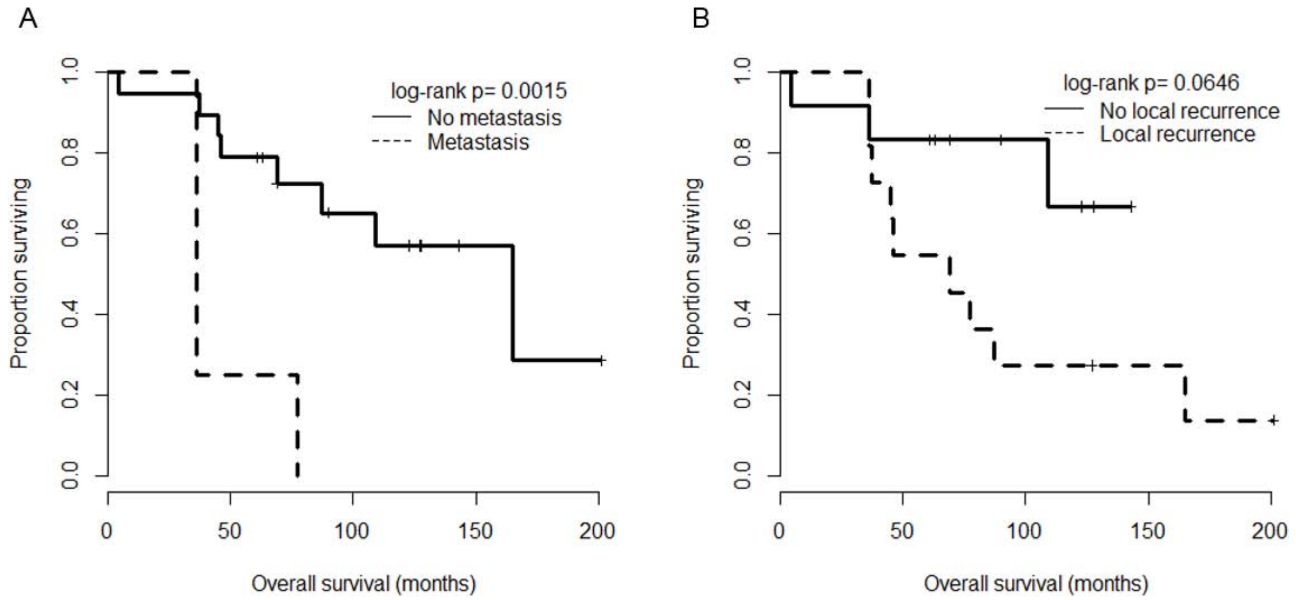


**Supplementary Figure S1: Clinical characteristics associated with overall survival.** Kaplan-Meier analysis comparing overall survival between chordoma patients with versus without metastatic disease **A.** and with versus without local recurrence **B.**
